# Aggressive end-of-life care in patients with gastrointestinal cancers – a nationwide study from Denmark

**DOI:** 10.2340/1651-226X.2024.41008

**Published:** 2024-11-24

**Authors:** Stine Gerhardt, Kirstine Skov Benthien, Suzanne Herling, Marie Villumsen, Peter-Martin Krarup

**Affiliations:** a Digestive Disease Center, Copenhagen University Hospital – Bispebjerg, Copenhagen, Denmark; b Palliative Care Unit, Copenhagen University Hospital, Hvidovre, Denmark; c REHPA – Danish Knowledge Centre for Rehabilitation and Palliative Care, Nyborg, Denmark; d The Neuroscience Center, Rigshospitalet, University of Copenhagen, Copenhagen, Denmark; e Centre for Clinical Research and Prevention, Copenhagen University Hospital – Frederiksberg, Copenhagen, Denmark

**Keywords:** Quality of life, palliative care, end-of-life care, neoplasms

## Abstract

**Background:**

Knowledge of determinants of aggressive end-of-life care is crucial to organizing effective palliative care for patients with gastrointestinal (GI) cancer.

**Purpose:**

This study aims to investigate the determinants of aggressive end-of-life care in patients with GI cancer.

**Methods:**

A national register-based cohort study using data from the Danish Register on Causes of Death, the Danish National Patient Register, and the Danish Palliative Database was the method of study employed.

**Participants/Setting:**

All Danish patients who died from GI cancers from 2010 to 2020 comprised the study setting.

**Results:**

There were 43,969 patients with GI cancers in the cohort, of whom 62% were hospitalized in the last 30 days of life, 41% of patients died in the hospital, 10% had surgery, 39% were subjected to a radiological examination during the last 30 days of life and 3% had antineoplastic treatment during the last 14 days of life. Among all types of GI cancers, pancreatic cancer was significantly associated with all outcomes of aggressive end-of-life care except surgery. Patients in specialized palliative care (SPC) had lower odds of receiving aggressive end-of-life care and dying in the hospital. We found that patients with comorbidity and those who were divorced had higher odds of being hospitalized at the end of life and dying in the hospital.

**Interpretation:**

Aggressive end-of-life care is associated with disease factors and socio-demographics. The potential to reduce aggressive end-of-life care is considerable in patients with GI cancer, as demonstrated by the impact of SPC. However, we need to address the needs of patients with GI cancer who do not receive SPC.

## Introduction

Preventing unnecessary and aggressive end-of-life care is essential for patients with incurable cancers near the end of life [1]. Indicators of aggressive end-of-life care include hospitalization, excessive healthcare use, chemotherapy administered close to the end of life, and dying in the hospital [2, 3]. Aggressive end-of-life care in patients with incurable cancer may compromise quality of life, increase suffering, and fail to provide significant benefits that align with patient values [2, 4]. Patients with gastrointestinal (GI) malignancies are at a high risk of receiving aggressive end-of-life care because of a severe symptom burden [5]. The incidence of GI cancers of the esophagus, stomach, pancreas, liver, bile duct, rectum, and colon are frequent worldwide with associated high cancer-related mortality rates, thus posing a significant organizational and economic burden to healthcare systems [6, 7]. It is crucial to identify patients at high risk of receiving aggressive end-of-life care to reduce burdensome overtreatment and to divert sparse healthcare resources to those who will profit the most.

Previous studies investigating determinants of aggressive end-of-life care in patients with cancer have reported that male gender, lower education levels, significant comorbidity and younger age were associated with a higher likelihood of aggressive end-of-life care [8, 9]. Conversely, being affiliated with specialized palliative care (SPC), which is the care provided by healthcare professionals where palliative care is the primary task, was associated with less aggressive end-of-life care [4, 8, 10, 11]. Merchant et al. found that a high proportion of patients with cancers of the colon, rectum, stomach, and esophagus were hospitalized (49%), received chemotherapy (8%), and died in the hospital (45%) in a Canadian population-based study [12]. However, there is a knowledge gap in understanding aggressive end-of-life care in patients with GI cancers, including futile surgery – and antineoplastic treatment, as well as the impact of concurrent admittance to SPC. Awareness of this complex interplay is crucial to improving timely decision-making and organizing palliative care in hospital departments outside of SPC for a group of patients with a complex symptom burden and a short life expectancy [7, 9].

Therefore, this study aimed to investigate aggressive end-of-life care determinants in patients with GI cancer.

## Methods

### Design and setting

This was a national register-based cohort study including all Danish patients dying from cancer in the esophagus, stomach, pancreas, liver, bile duct, rectum, and colon from 2010 to 2020, with at least one registered contact with a Danish hospital. In Denmark, all patients with cancer have free access to diagnostics, treatment, and palliative care, as taxes finance the healthcare system.

### Data sources

Patients in the Danish Register of Causes of Death were identified and linked to the cohort of the National Patient Register for data on hospital admissions, healthcare use, and diagnoses for comorbidity status using the unique personal identification issued to all Danish citizens. We derived data on age, gender, education level, and marital status from Statistics Denmark. The place of death was extracted from the Danish Register on Causes of Death. Finally, we linked all patients to the Danish Palliative Database for data on admission to SPC (yes/no).

### Study outcomes

The primary outcome was hospital admissions, defined as one overnight stay of at least 8 hours (yes/no) within the last 30 days of life. Secondary outcomes included surgery within the last 30 days of life, defined as any surgery except for procedures with purely palliative intent, such as stent placements, and radiological examinations, described as a CT scan (Computer Tomography scan), a PET CT scan (Positron Emission Tomography scan), and an MRI scan (Magnetic Resonance Imaging), within the last 30 days of life (yes/no), antineoplastic treatment, defined as chemotherapy or immune therapy, within the last 14 days of life (yes/no), and death at the hospital (yes/no).

### Explanatory variables

Age, gender, cancer diagnosis, education level, marital status, comorbidity, region of residence, and admission to SPC were included as explanatory variables.

Age was categorized into three groups: 18–64 years, 65–79 years, and 80 years and above, respectively. Education level was categorized as (1) primary and lower secondary school, (2) upper secondary and post-secondary (vocational), (3) short tertiary and bachelor’s level, (4) Master’s level or above, and (5) not classified [13]. We grouped marital status as (1) widow(er), (2) divorced/separated, (3) married/living with a partner, and (4) never married. Regions of residence were the North Denmark Region, the Central Denmark Region, the Region of Southern Denmark, Region Zealand, and the Capital Region of Denmark as registered in Statistics Denmark. The five regions have different healthcare services organizations, life expectancies ranging from 80.5 years to 81.7, and vary in population size, density, and hospital proximity [14]. The severity of comorbidity was calculated as a sum of weighted scores for concurrent diseases according to the Charlson Comorbidity Index (CCI) and based on ICD-10 [15] from the hospitalization history of any contact from 1998, excluding cause of death. The final score was grouped as score <2 or ≥2, where a higher score indicates more severe comorbidity, and finally, we included admission to SPC (yes/no). In Denmark, SPC is provided in hospital units and hospices in inpatient and outpatient settings. Esophagus-, stomach-, pancreatic-, liver-, and bile duct cancers are referred to as upper GI cancer.

### Statistical analyses

Patient characteristics were reported in numbers and percentages for each subgroup of GI cancers included in the cohort.

Crude and adjusted logistic regression analyses were applied to estimate the association with all outcomes and reported in odds ratios (OR) and corresponding 95% confidence intervals (95% CI). The adjusted models included age, gender, comorbidity score, education level, marital status, region of residence, and admission to SPC. We performed sensitivity analyses for patients with pancreatic cancer to investigate differences between the group of patients who were affiliated with SPC and those who were not. Analyses were performed in SAS 9.4.

## Results

The cohort consisted of 43,969 patients with GI cancers of the colon (28%), pancreas (23%), rectum (15%), esophagus (12%), stomach (7%), bile ducts cancers (7%), liver (6%), and ‘other gastrointestinal cancers’ (2%) of whom 50% were affiliated with SPC. Most patients were male (57%) and between 65 and 79 years old (48%). Table 1 shows the characteristics of the cohort.

**Table 1:** Characteristics of the study population.

Variables	CANCER DIAGNOSIS																	
	All *N* = 43,969		Colon *n* = 12,222 (28%)		Pancreas *n* = 10,327 (23%)		Rectum *n* = 6,511 (15%)		Esophagus *n* = 5,444 (12%)		Stomach *n* = 2,995 (7%)		Bile ducts *n* = 2,982 (7%)		Liver *n* = 2,590 (6%)		Other *n* = 898 (2%)	
	*n*	%	*n*	%	*n*	%	*n*	%	*n*	%	*n*	%	*n*	%	*n*	%	*n*	%
**Gender**																		
Male	25,141	57	6,049	50	530	52	3,965	61	4,071	75	1,877	63	1,376	46	2,010	78	486	54
Female	18,673	43	6,122	50	4,994	48	2,515	39	1,349	25	1,105	37	1,599	54	577	22	412	46
**Age**																		
18–64	9,303	21	1,972	16	2,218	21	1,283	20	1,571	29	734	25	646	22	678	26	186	21
65–79	21,221	48	5,243	43	5,542	54	2,986	46	2,718	50	1,355	45	1,469	49	1,437	56	471	52
80+	13,460	31	5,007	41	2,567	25	2,242	34	1,155	21	906	30	867	29	475	18	241	27
**Charlson comorbidity Index**																		
<2	27,223	62	7,578	62	6,571	64	4,199	65	3,400	63	1,912	64	1,936	65	1,146	44	481	54
≥2	16,662	38	4,610	38	3,744	36	2,289	35	2,037	37	1,079	36	1,042	35	1,444	56	417	46
**Education level**																		
Primary, lower secondary	17,649	40	5,016	41	3,990	39	2,653	41	2,079	38	1,330	44	1,236	42	1,000	39	336	37
Upper, post secondary (vocational)	16,844	38	4,374	36	4,031	39	2,481	38	2,277	42	1,074	36	1,085	36	1,138	44	361	40
Short tertiary, Bachelor’s	5,815	13	1,662	14	1,523	15	800	12	675	12	347	12	389	13	276	11	139	16
Masters’ level, above	1,813	4	509	4	475	5	253	4	211	4	110	4	131	4	85	3	38	4
**Marital status**																		
Married/partner	10,151	23	3,553	29	2,167	21	1,588	25	869	16	648	22	755	25	398	15	173	19
Widow	6,293	14	1,527	13	1,546	15	918	14	906	17	384	13	382	13	506	20	124	14
Divorced/separated	23,203	53	6,038	50	5,751	56	3,360	52	2,942	54	1,661	56	1,588	53	1,342	52	521	58
Not married	4,100	9	1,037	9	820	8	604	9	696	13	284	10	245	8	336	13	78	9
**Region of residence**																		
Capital Region of Denmark	12,265	28	3,464	28	3,038	29	1,701	26	1,602	30	663	22	788	27	802	31	207	23
Region of Zealand	7,564	17	2,132	17	1,732	17	1,165	18	1,024	19	429	14	480	16	449	17	153	17
North Denmark Region	4,823	11	1,281	11	1,185	11	720	11	430	8	495	17	331	11	275	11	106	12
Region of South Denmark	9,973	23	2,729	22	2,261	22	1,501	23	1,258	23	711	24	730	24	544	21	239	27
Central Region Denmark	9,210	21	2,583	21	2,080	20	1,407	22	1,107	20	686	23	642	22	514	20	191	21
**Specialized palliative care**																		
Admitted	22,118	50	5,451	45	5,971	58	3091	48	2,908	53	1,579	53	1,519	51	1,115	43	484	54

‘Other digestive cancers’: cancers of the ileum and cancers defined as ‘other digestive cancers’ in the Danish Register on Causes of Death.

### Hospitalization

Overall, 62% of all patients in the cohort were hospitalized within the last 30 days of life. Patients with upper GI cancers had higher odds of being hospitalized in the last 30 days of life compared to patients with colorectal cancer (Table 2). Patients with pancreatic cancer (OR 1.30 (95% CI 1.23–1.37)) and patients with cancer of the bile ducts (OR 1.23 (95% CI 1.13–1.34)) had the highest risk compared to patients with cancer in the colon. We also found that patients with liver cancer had a greater risk of being hospitalized (OR 1.13 (95% CI 1.03–1.24)) compared to patients with colon cancer, while patients with cancer in the rectum had lower odds of hospitalization within the last 30 days (OR 0.79 (95% CI 0.79–0.84)). Other risk factors for being hospitalized the last 30 days of life included Charlson Comorbidity Index ≥2 (OR 1.05 (95% CI 1.01–1.20)) and being divorced or separated from a partner compared to being married (OR 1.22 (95% CI 1.15–1.28)) and this was associated with hospitalization in the last 30 days of life. Patients affiliated with SPC were less likely to be hospitalized at the end of life than patients who were not (OR 0.68 (95% CI 0.65–0.71)). Region of residence was associated with hospitalization. Patients living in the Region of South Denmark had a decreased risk of being hospitalized (OR 0.93 (95% CI 0.88–0.98)). The same was true for patients living in the Central Denmark Region (OR 0.85 (95% CI 0.80–0.90)) compared to patients living in the Capital Region of Denmark.

**Table 2:** Hospitalizations and deaths at the hospital.

Explanatory variables *N* = 43,969	Hospitalized		Hospitalized OR (95%CI)				Death at hospital		Death at hospital OR (95%CI)			
			Crude		Adjusted				Crude		Adjusted	
	*n*	%	OR	95%CI	OR	95%CI	*n*	%	OR	95%CI	OR	95%CI
**Gender**												
Male	16,185	64	Ref		Ref		10,793	43	Ref		Ref	
Female	10,856	58	0.77	0.74–0.80	0.84	0.80–0.87	7,176	38	0.83	0.80–0.86	0.95	0.91–0.99
**Age (years)**												
18–64	6,095	65	Ref		Ref		4,303	46	Ref		Ref	
65–79	13,605	64	0.94	0.89–0.99	0.85	0.80–0.89	9,314	44	0.89	0.85–0.94	0.77	0.74–0.82
80+	7,379	55	0.64	0.60–0.67	0.60	0.56–0.63	4,384	32	0.55	0.52–0.58	0.43	0.41–0.46
**Cancer diagnosis**												
Colon	7,221	59	Ref		Ref		4,874	40	Ref		Ref	
Rectum	3,551	55	0.83	0.78–0.88	0.79	0.75–0.84	2,398	37	0.88	0.83–0.94	0.86	0.80–0.91
Esophagus	3,429	63	1.18	1.10–1.26	1.05	0.98–1.12	2,394	44	1.18	1.11–1.26	1.07	0.99–1.15
Stomach	1,867	62	1.15	1.06–1.25	1.08	0.99–1.17	1,167	39	0.96	0.86–1.04	0.98	0.90–1.07
Pancreas	3,806	66	1.34	1.27–1.41	1.30	1.23–1.37	4,390	43	1.11	1.05–1.17	1.10	1.04–1.16
Liver	1,710	65	1.35	1.23–1.47	1.13	1.03–1.24	1,161	39	1.21	1.12–1.33	0.97	0.89–1.07
Bile ducts	1,928	63	1.27	1.17–1.38	1.23	1.13–1.34	1,210	41	1.03	0.95–1.11	1.01	0.93–1.10
Other	567	63	1.19	1.03–1.37	1.11	0.97–1.29	407	45	1.24	1.09–1.43	1.26	1.09–1.45
**Charlson comorbidity index**												
<2	16,688	61	Ref		Ref		10,976	40	Ref		Ref	
≥2	10,391	62	1.05	10.1–1.09	1.05	1.01–1.20	7,025	42	1.08	1.04–1.12	1.07	1.03–1.12
**Education level**												
Primary, lower secondary	10,609	60	Ref		Ref		6,783	38	Ref		Ref	
Upper secondary, post-secondary (vocational)	10,725	64	1.16	1.11–1.22	1.05	1.00–1.10	7,299	43	1.26	1.17–1.28	1.10	1.05–1.15
Short tertiary, bachelor’s level	3,617	62	1.09	1.03–1.16	1.02	0.96–1.09	2,405	41	1.13	1.06–1.20	1.05	0.98–1.12
Master’s level or above	1,122	62	1.08	0.98–1.19	0.95	0.86–1.05	870	48	1.48	1.34–1.63	1.23	1.11–1.36
**Marital status**												
Married/partner	5,611	55	Ref		Ref		3,512	38	Ref		Ref	
Widow	3,857	61	1.28	1.20–1.37	1.04	0.97–1.12	2,710	43	1.43	1.34–1.53	1.15	1.07–1.26
Divorced/separated	15,028	65	1.49	1.42–1.56	1.22	1.15–1.28	9,909	43	1.41	1.34–1.48	1.17	1.11–1.24
Not married	2,504	61	1.27	1.18–1.37	0.96	0.88–1.04	1,803	44	1.48	1.38–1.60	1.05	0.96–1.14
**Region of residence**												
Capital Region of Denmark	7,723	63	Ref		Ref		6,574	54	Ref		Ref	
Region of Zealand	4,709	62	0.97	0.91–1.03	0.96	0.91–1.02	2,957	39	0.56	0.52–0.59	0.54	0.51–0.57
North Denmark Region	2,957	61	0.93	0.87–1.00	0.98	0.92–1.06	1,710	35	0.48	0.44–0.51	0.53	0.49–0.57
Region of South Denmark	6,167	62	0.95	0.90–1.00	0.93	0.88–0.98	3,764	38	0.52	0.50–0.55	0.49	0.46–0.52
Central Denmark Region	5,446	59	0.85	0.80–0.90	0.85	0.80–0.90	2,934	32	0.41	0.38–0.43	0.39	0.37–0.41
**Specialized palliative care**												
No	14,089	65	Ref		Ref		10,671	49	Ref		Ref	
Yes	12,976	59	0.77	0.74–0.80	0.68	0.65–0.71	7,315	33	0.51	0.49–0.53	0.42	0.40–0.43

Crude and adjusted logistic regression analysis adjusted for age, gender, cancer diagnoses, Charlson Comorbidity Index, education level, marital status, admission to specialized palliative care, and region of residence.

### Death at the hospital

Forty-one per cent of all patients investigated died in the hospital. The adjusted analyses revealed that age above 80 was associated with decreased odds of dying in the hospital (OR 0.43 (CI 95% 0.41–0.46)). Patients with cancer of the pancreas (OR 1.10 (95% CI 1.04–1.16)) had higher odds of dying in the hospital compared to patients with colon cancer or rectal cancer. Of all patients with pancreatic cancer in the cohort, 43% died in the hospital, and for patients with cancer in the esophagus, the proportion was 44%. Greater comorbidity was associated with death in the hospital (OR 1.07 (95% CI 1.03–1.12)). Divorced or separated patients (OR 1.17 (95% CI 1.11–1.24)) and widowed (OR 1.15 (95% CI 1.07–1.26)) had higher odds of dying in the hospital compared to patients who were married or cohabitating patients. Patients affiliated with SPC had lower odds of dying in the hospital (OR 0.42 (95% CI 0.40–0.43)). Results are shown in Table 2. Region of residence was associated with death in the hospital, with the lowest risk of dying in the hospital for patients living in the Central Denmark Region (OR 0.39 (95% CI 0.37–0.41)) compared to the Capital Region of Denmark.

### Factors associated with surgery, radiological examinations, and antineoplastic treatment

In this cohort, 10% of the patients had surgery within the last 30 days of life. In the adjusted analyses, being above 80 years old (OR 0.71 (95% CI 0.64–0.79)) and suffering from upper GI cancers were associated with surgery within the last 30 days of life, while the lowest risk was observed in patients with liver cancer (OR 0.33 (95% CI 0.28–0.40)) and bile duct cancers (OR 0.41 (95% CI 0.35–0.48)) compared to patients with a colon cancer diagnosis. Furthermore, patients affiliated with SPC had a decreased risk of surgery in the last 30 days of life (OR 0.38 (95% CI 0.35–0.41)). In this cohort, patients with education of ‘Master’s level and above’ (compared to ‘primary and lower secondary’) (OR 1.33 (95% CI 1.13–1.56)) and a comorbidity score of 2 or above (OR 1.10 (95% CI 1.04–1.18)) were associated with increased risk of surgery.

In total, 39% of the patients were subjected to a CT, PET CT, or MRI examination within the last 30 days of life (Table 3). Pancreatic cancer (OR 1.12 (95% CI 1.06–1.19)), being divorced (compared to married) (OR 1.12 (95% CI 1.06–1.18)), and having all education levels other than ‘primary and lower secondary’ education were associated with higher odds of receiving a CT, PET CT, or MRI examination the last 30 days of life (Table 3). Patients with rectal cancer (OR 0.77 (95% CI 0.72–0.82)) and esophagus cancer (OR 0.80 (95% CI 0.74–0.86)) had lower odds of receiving a CT, PET CT, or MRI examination compared to patients with colon cancer as well as patients admitted to SPC (OR 0.47 (95% CI 0.45–0.49)), who had lower odds of receiving a CT, PET CT, and MRI examination the last 30 days of life compared to patients not admitted to SPC.

**Table 3:** Surgery, radiological examinations, and antineoplastic treatment.

Explanatory variables	Surgery		Radiological examinations										Antineoplastic treatment					
	*N*	%	OR (CI 95%)				*N*	%	OR (CI 95%)				*N*	%	OR (CI 95%)			
			Crude		Adjusted				Crude		Adjusted				Crude		Adjusted	
			OR		CI 95%				OR		CI 95%				OR		CI 95%	
**Gender**																		
Male	2,603	10	Ref		Ref		10,279	41	Ref		Ref		797	3	Ref		Ref	
Female	1,776	9	0.91	0.85–0.97	0.94	0.90–0.98	7,056	38	0.87	0.84–0.91	0.94	0.90–0.98	457	2	0.97	0.93–1.01	0.85	0.75–0.97
**Age (years)**																		
18–64	863	9	Ref		Ref		4,190	45	Ref		Ref		381	4	Ref		Ref	
65–79	2,180	10	0.89	0.82–0.97	0.93	0.85–1.17	8,833	42	0.74	0.71–0.78	0.75	0.71–0.79	750	3	0.86	0.76–0.97	0.69	0.61–0.79
80+	1,351	10	0.97	0.91–1.05	0.71	0.64–0.79	4,312	32	0.57	0.54–0.60	0.45	0.42–0.48	123	1	0.21	0.18–0.26	0.16	0.13–0.20
**Cancer diagnosis**																		
Colon	1,670	14	Ref		Ref		4,880	40	Ref		Ref		343	3	Ref		Ref	
Rectum	744	11	0.82	0.75–0.90	0.81	0.74–0.89	2,264	35	0.80	0.75–0.85	0.77	0.72–0.82	190	1	1.04	0.87–1.25	0.97	0.81–1.16
Esophagus	476	9	0.61	0.54–0.67	0.60	0.54–0.68	2,016	37	0.89	0.83–0.95	0.80	0.74–0.86	118	2	0.77	0.62–0.95	0.60	0.49–0.75
Stomach	318	11	0.75	0.66–0.85	0.76	0.66–0.86	1,176	39	0.96	0.86–1.04	0.93	0.86–1.02	61	2	0.72	0.55–0.95	0.67	0.51–0.89
Pancreas	721	7	0.47	0.43–0.52	0.50	0.45–0.55	4,448	43	1.12	1.08–1.20	1.12	1.06–1.19	425	4	1.49	1.29–1.72	1.38	1.19–1.60
Liver	147	6	0.38	0.32–0.45	0.33	0.28–0.40	935	36	0.85	0.78–0.96	0.69	0.63–0.76	31	1	0.42	0.29–0.61	0.30	0.21–0.44
Bile ducts	181	6	0.41	0.35–0.48	0.41	0.35–0.48	1,234	41	1.07	0.98–1.15	1.03	0.95–1.12	70	2	0.83	0.64–1.08	0.76	0.60–0.99
Other	122	13	0.99	0.82–1.21	1.00	0.82–1.23	382	42	1.12	0.97–1.28	1.07	0.93–1.23	16	2	0.63	0.38–1.04	0.60	0.36–1.00
**Charlson Comorbidity Index**																		
<2	2,625	10	Ref		Ref		10,798	40	Ref		Ref		885	3	Ref		Ref	
≥2	1,754	10	1.10	1.03–1.18	1.10	1.04–1.18	6,537	39	0.98	0.95–1.02	1.02	0.97–1.06	369	2	0.68	0.60–0.76	0.72	0.63–0.82
**Education level**																		
Primary, lower secondary	1,757	10	Ref		Ref		6,617	37	Ref		Ref		418	2	Ref		Ref	
Upper, post–secondary (vocational)	1,714	10	1.03	0.96–1.10	1.08	1.01–1.17	6,944	41	1.17	1.12–1.22	1.11	1.06–1.16	543	3	1.38	1.21–1.57	1.17	1.02–1.34
Short tertiary, bachelor’s	544	9	0.93	0.84–1.03	1.05	0.95–1.17	2,406	41	1.18	1.11–1.25	1.16	1.08–1.23	207	3	1.52	1.29–1.81	1.37	1.15–1.64
Masters’ level, above	206	11	1.16	1.00–1.35	1.33	1.13–1.56	755	42	1.19	1.08–1.32	1.15	1.04–1.28	59	3	1.40	1.05–1.83	1.18	0.89–1.58
**Marital status**																		
Married/partner	1,057	10	Ref		Ref		3,501	34	Ref		Ref		171	2	Ref		Ref	
Widow	588	9	0.89	0.80–0.99	0.92	0.82–1.03	2,492	40	1.25	1.17–1.33	1.02	0.95–1.10	193	3	0.89	0.80–0.99	1.08	0.86–1.34
Divorced/Separated	2,322	10	0.96	0.89–1.03	0.97	0.89–1.06	9,640	41	1.35	1.29–1.42	1.12	1.06–1.18	763	3	0.96	0.89–1.03	1.19	0.99–1.43
Not married	406	9	0.95	0.84–1.07	0.93	0.82–1.07	1,670	41	1.30	1.21–1.41	0.97	0.90–1.06	124	3	0.95	0.84–1.07	0.94	0.73–1.21
**Region of residence**																		
Capital Region of Denmark	1,134	9	Ref		Ref		4,909	40	Ref		Ref		375	3	Ref		Ref	
Region of Zealand	685	9	0.98	0.89–1.08	0.98	0.89–1.09	2,891	38	0.93	0.88–0.99	0.93	0.88–0.99	279	4	1.21	1.04–1.42	1.20	1.02–1.41
North Denmark Region	507	10	1.15	1.03–1.29	1.34	1.19–1.50	1,933	40	1.13	1.05–1.21	1.29	1.05–1.21	100	2	0.67	0.54–0.84	0.78	0.62–0.98
Region of South Denmark	1,108	11	1.23	1.12–1.34	1.18	1.08–1.29	4,038	40	0.99	0.94–1.05	0.99	0.94–1.05	242	2	0.79	0.67–0.93	0.75	0.63–0.88
Central Denmark Region	941	10	1.12	1.02–1.22	1.12	1.02–1.23	3,532	38	0.93	0.88–0.99	0.93	0.88–0.99	255	3	0.90	0.77–1.06	0.90	0.76–1.05
**Specialized palliative care**																		
No	3,048	14	Ref		Ref		10,086	46	Ref		Ref		914	4	Ref		Ref	
Yes	1,331	6	0.39	0.37–0.42	0.38	0.35–0.41	7,249	32	0.57	0.54–0.59	0.47	0.45–0.49	340	1	0.36	0.31–0.41	0.26	0.23–0.29

Crude and adjusted logistic regression analysis adjusted for age, gender, cancer diagnoses, Charlson Comorbidity Index, education level, marital status, admission to specialized palliative care, and region of residence.

Of the patients in the cohort, 3% received antineoplastic treatment within the last 14 days of life.

A pancreatic cancer diagnosis was associated with receiving antineoplastic treatment in the last 14 days of life compared to all other cancer types (OR 1.38 (95% CI 1.19–1.60)). Female gender (OR 0.85 (95% CI 0.75–0.97)) and comorbidity (OR 0.72 (95% CI 0.63–0.82)) were associated with lower odds of receiving antineoplastic treatment, as well as a short tertiary, bachelor’s education level (OR 1.37 (95% CI 1.15–1.64)) and living in the Region of Zealand (OR 1.20 (95% CI 1.02–1.41)).

### Sensitivity analyses

In the cohort, 58% of patients with pancreatic cancer were affiliated with SPC. The sensitivity analyses revealed that 62% of the SPC-affiliated patients with pancreatic cancer were hospitalized, compared to 71% of non-SPC-affiliated patients. Additionally, 35% of SPC-affiliated patients died at the hospital, while 54% of non-affiliated patients with pancreatic cancer did. Moreover, 2.5% of SPC-affiliated patients received antineoplastic treatment, whereas 6% of non-affiliated patients did. Thirty-six per cent of patients with pancreatic cancer affiliated with SPC received a radiological examination, while 53% of non-SPC affiliated patients with pancreatic cancer did.

## Discussion

### Main findings

In this study of patients with GI cancers, a high proportion were hospitalized near the end of life, with pancreatic cancer patients having a higher risk of aggressive end-of-life care. Affiliation with SPC was associated with a reduced risk, highlighting that SPC is relevant and effective. In our study cohort, 50% of the patients were affiliated with SPC, which is similar to the 50% rate in the general cancer population of Denmark [16].

Over half of the patients in the cohort were hospitalized in the last 30 days of life, defined as an overnight stay of at least 8 hours. Merchant et al. discovered that just under half (49.3%) of a Canadian population-based sample of patients with GI cancer were hospitalized in the last 30 days of life [12]. Miesfeldt et al. investigated aggressive end-of-life care for colon and pancreatic cancer patients and found that 61.3% were hospitalized in the last month of life [8]. Although the populations in our study are different from those of the Miesfeldt study, it is worth noting that the authors also investigated patients with pancreatic cancer in their cohort and found similarly high proportions of hospitalizations in the last 30 days of life [8]. Other studies have investigated aggressive end-of-life care, such as multiple hospitalizations within the last 30 days of life or rehospitalizations during different time intervals [9, 11, 17, 18], and are not directly comparable to the present study. In the present study, pancreatic-, bile duct- and liver cancer were associated with an increased risk of being hospitalized. One possible explanation is that these cancers can be particularly aggressive, with a severe symptom burden possibly leading to hospitalizations [11, 17, 19–21]. Another explanation may be the lack of conversations in hospital departments outside SPC about end-of-life preferences. These conversations are referred to as advance care planning (ACP), an important component of SPC. ACP involves structured conversations between healthcare professionals and patients about their preferences for end-of-life treatment and care [22]. Although ACP is not explicitly analyzed in our study it may contribute to the reduced aggressive end-of-life care observed in GI cancer patients affiliated with SPC. Other potentially effective components of SPC are symptom management and home-based follow-up. The results from our study build upon the effects of SPC found in previous research investigating aggressive end-of-life care outcomes [4, 11, 23–25]. An RCT by Temel et al. investigating early SPC versus standard oncological care showed that 33% of patients receiving SPC received aggressive end-of-life care versus 54% in the standard oncological care group [25].

This study found a high rate of hospital deaths, which is consistent with previous research demonstrating similar in-hospital mortality rates of 44.6% [12] and 37.9% [4]. Although we lack information on patients’ preferred place of death, dying in hospitals may not align with their wishes [26, 27]. Dying in the hospital can also be a result of a lack of ACP [22].

This study confirms the findings of previous studies: female and older patients were less likely to experience hospitalization and die in the hospital, and patients with pancreatic cancer, greater comorbidity, higher education than ‘primary and lower secondary’ education, and those who were divorced or widowed had higher odds of being hospitalized at the end of life and die in the hospital [9, 12, 8, 17].

The characteristics of patients who underwent surgery within 30 days of death were similar to those associated with other aggressive end-of-life care outcomes found in previous studies [8, 28, 29]. Patients with a higher level of education and greater comorbidity had higher odds of undergoing end-of-life surgery, while those above 80 years and of female gender were associated with lower odds [9]. Patients with upper GI cancer and rectal cancer had a lower likelihood of undergoing surgery within the last 30 days of life compared to patients with colon cancer. This is the first study to report on surgery as a dependent variable in a large cohort of GI cancers within the last 30 days of life. This finding is likely related to the risk of bowel obstruction in patients with colon cancer caused by tumor blockage or carcinomatosis [30]. The primary focus in a surgical hospital department is often relieving obstructive symptoms through emergency surgical intervention. Surgery can be successful, but only briefly for many patients [30, 31]. A systematic review showed high rates of 30-day postoperative mortality, complications, and hospital readmissions in patients undergoing open or laparoscopic surgery for malignant bowel obstruction [31]. Thus, clinicians need to facilitate decision-making that is aligned with patients’ goals and preferences for treatment and care. Additionally, clinicians should communicate the high risk of adverse outcomes following surgery, which may compromise the quality of palliative care [30, 32].

In the current study, 3% of patients received antineoplastic treatment within the last 14 days of life. This aligns with previous studies, which reported rates of 4.2% and 7.1% in studies by Benthien et al. and Massa et al. respectively. These findings are consistent with the proposed quality metrics for chemotherapy rates, which should be below 10%, as described by Earle et al. [33–35].

Pancreatic cancer showed significant associations with all aspects of aggressive end-of-life care, except for surgery, in the analyses that accounted for SPC affiliation. The same findings were demonstrated by Khan et al. [17], underlining that this subgroup within the GI cancer population is particularly at risk of receiving aggressive end-of-life care. Pancreatic cancer is characterized by a severe physical and psychological symptom burden [36–38], resulting in complex palliative care needs requiring SPC [21, 39]. Our sensitivity analyses revealed that patients with pancreatic cancer affiliated with SPC had a 20% lower in-hospital mortality rate than those who were not. The rate of hospitalization at the end of life was 10% lower for pancreatic cancer patients affiliated with SPC compared to the patients who were not. However, there was still a high hospitalization rate of 62% for SPC-affiliated patients with pancreatic cancer at the end of life, highlighting the severe symptom burden and need for SPC. Adsersen et al. found that patients with pancreatic cancer and stomach cancer had the highest odds of admittance to SPC in a large Danish population-based study of all cancer deaths from 2010 to 2012; this was also confirmed by Miesfeldt et al. [8, 39].

Patients living in all regions of residence other than the Capital Region of Denmark had fewer hospital deaths, which might be caused by the longer distance to hospitals in regions other than the Capital Region of Denmark and lower hospital bed capacity [40].

### Perspective

Hospital departments and clinicians who are responsible for the treatment and palliative care of patients with GI cancers should be particularly attentive to patients with upper GI cancers, younger patients, males, divorced or widowed patients, and patients with complex palliative care needs. Patients with pancreatic cancer might benefit from tailored interventions with close follow-up and early referral to SPC. Patients with cancers of the colon and malignant bowel obstruction should be offered ACP early in their disease trajectory with a focus on adverse treatment outcomes following potential surgery. It is essential to prioritize interventions that promote ACP and shared decision-making when developing generalist PC interventions in hospital departments to avoid aggressive end-of-life care.

### Strengths and limitations

A strength of this study is the large cohort, including all patients with GI cancers, several variables of aggressive end-of-life care, and affiliation with SPC from validated Danish registers and databases with close to 100% completeness. Limitations include the lack of data on patients’ preferred place of death, which is not available in Danish registers. Other potential confounders not contained in this study are contacts with general practitioners. Another limitation is the inability to differentiate between patients who died in a SPC department or a non-SPC hospital department. An essential limitation is that the observed effects of SPC on end-of-life outcomes may be confounded by indication, as patients referred to SPC may be more inclined to engage in ACP and demand less aggressive treatment.

## Conclusion

Aggressive end-of-life care is associated with disease factors such as cancer diagnosis and comorbidity that may determine indication, but also by socio-demographics, which should not affect treatment indication but may reflect healthcare literacy, support needs, and patient demands of the healthcare system. The potential to reduce aggressive end-of-life care is considerable in patients with GI cancer, as demonstrated by the impact of SPC. Evidence-based interventions to improve healthcare decision-making and ACP for patients with GI cancer not receiving SPC are strongly warranted.

## Data Availability

The data is not publicly available due to legal restrictions.
